# Commentary: How Cells Can Control Their Size by Pumping Ions

**DOI:** 10.3389/fcell.2017.00072

**Published:** 2017-08-21

**Authors:** Igor A. Vereninov, Valentina E. Yurinskaya, Alexey A. Vereninov

**Affiliations:** ^1^Saint Petersburg State Polytechnic University Saint Petersburg, Russia; ^2^Institute of Cytology (RAS) Saint Petersburg, Russia

**Keywords:** cell water regulation, impermeant anions, ion transport, potassium, chloride channels, chloride exchangers, sodium-hydrogen exchanger

Intracellular water typically occupies about 80% of the total cell volume. The very important theoretical article by Alan Kay calls attention to the roles of impermeant intracellular osmolytes and ion pumping in the regulation of cell water. Theoretical predictions presented by A. Kay agree with available experimental data. In our study of U937 cells going into apoptosis (Yurinskaya et al., 2011), only 56–72% of the observed volume loss could be explained by the efflux of monovalent ions; a significant portion of the lost osmolytes must have been the “impermeant intracellular anions.” No pumping of chloride was assumed in the calculations presented by Kay. In our opinion, this is a significant shortcoming of the model because the role of chloride channels and transporters in cell water and chloride regulation has been proved beyond doubt (Hoffmann et al., 2009, 2015; Voipio et al., 2014). Our recent studies show how the major chloride co- and counter-transporters and channels can determine chloride disequilibrium distribution across the membrane and the electrochemical potential differences under various conditions (Vereninov et al., 2014, 2016). We have developed a computational software to determine the fluxes of monovalent ions in cells of various kinds, with membrane potentials from −5 to −90 mV and the intracellular K^+^/Na^+^ ratios between 0.2 and 9, i.e., for the entire range of values encountered in real animal cells. The modeling of cell water balance as a function of various transport rates shows, in particular, that the coupled operation of Na/H and Cl/HCO_3_ exchangers and chloride channels are much more powerful regulators of water balance than NKCC, KCC cotransporters, or the Na/K ATPase pump. The role of Na/H and Cl/HCO_3_ exchangers should be stressed especially. It is known since the1980th (see e.g., Grinstein et al., 1988). Lew was the first who includes Na-Cl cotransport in the calculation of the flux balance in cells. Lew stressed that “reticulocytes, unlike mature RBCs, need Na^+^-dependent anion influx transporters to maintain volume stability” (Lew et al., 1991, p. 105). Our computation of the monovalent ion flux balance was performed assuming the values of rate coefficients obtained on proliferating human lymphoid cells U937, which may serve as a prototype of cells with intermediate values of the membrane potential and of the K^+^/Na^+^ ratio. The computational modeling showed that it is the rate of Cl^−^ gain that determines the kinetics of cell ion and water changes caused by blocking the Na/K ATPase pump and that the rate of Cl^−^ redistribution may be very slow in spite of the fast Cl^−^/Cl^−^ exchange. As a result, the disturbance caused by the Na/K pump inhibition may initially proceed as a nearly equivalent exchange of K^+^ for Na^+^ with no cell swelling. This computational prediction was fully confirmed in experiments with U937 cells.

## Author contributions

All authors listed have made a substantial, direct and intellectual contribution to the work, and approved it for publication.

### Conflict of interest statement

The authors declare that the research was conducted in the absence of any commercial or financial relationships that could be construed as a potential conflict of interest.

## References

[B1] Grinstein S. Garcia-Soto J. Mason M. J. (1988). Proton passage across cell membranes. Ciba Found. Symp. 139, 70–90.2849531

[B2] Hoffmann E. K. Belinda H. Sørensen B. H. Sauter D. P. Lambert I. H. (2015). Role of volume-regulated and calcium-activated anion channels in cell volume homeostasis, cancer and drug resistance. Channels 9, 380–396. 10.1080/19336950.2015.1089007 26569161 PMC4850047

[B3] Hoffmann E. K. Lambert I. H. Pedersen S. F. (2009). Physiology of cell volume regulation in vertebrates. Physiol. Rev. 89, 193–277. 10.1152/physrev.00037.2007 19126758

[B4] Lew V. L. Freeman C. J. Ortiz O. E. Bookchin R. M. (1991). A mathematical model of the volume, pH, and ion content regulation in reticulocytes. Application to the pathophysiology of sickle cell dehydration. J. Clin. Invest. 87, 100–112. 1985088 10.1172/JCI114958 PMC295002

[B5] Vereninov I. A. Yurinskaya V. E. Model M. A. Lang F. Vereninov A. A. (2014). Computation of pump-leak flux balance in animal cells. Cell. Physiol.Biochem. 34, 1812–1823, 10.1159/000366382 25502638

[B6] Vereninov I. A. Yurinskaya V. E. Model M. A. Vereninov A. A. (2016). Unidirectional flux balance of monovalent ions in cells with Na/Na and Li/Na exchange: experimental and computational studies on lymphoid U937 cells. PLoS ONE 11:e0153284. 10.1371/journal.pone.0153284 27159324 PMC4861346

[B7] Voipio J. Boron W. F. Jones S. W. Hopfer U. Payne J. A. Kaila K. (2014). Comment on “Local impermeant anions establish the neuronal chloride concentration.” Science 345:1130 10.1126/science.1252978 25190787

[B8] Yurinskaya V. E. Rubashkin A. A. Vereninov A. A. (2011). Balance of unidirectional monovalent ion fluxes in cells undergoing apoptosis: why does Na^+^/K^+^ pump suppression not cause cell swelling? J. Physiol. (Lond.) 589(Pt 9), 2197–2211. 10.1113/jphysiol.2011.207571 21486767 PMC3098698

